# Preventing and Managing Conflict of Interest in Nutrition Policy: Lessons for Alcohol Control Comment on "Towards Preventing and Managing Conflict of Interest in Nutrition Policy? An Analysis of Submissions to a Consultation on a Draft WHO Tool"

**DOI:** 10.34172/ijhpm.2020.161

**Published:** 2020-08-19

**Authors:** Katherine Severi

**Affiliations:** Institute of Alcohol Studies (IAS), London, UK.

**Keywords:** Alcohol Policy, Conflicts of Interest, Alcohol Industry, Health Governance

## Abstract

Ralston et al present an analysis of policy actor responses to a draft World Health Organization (WHO) tool to prevent and manage conflicts of interest (COI) in nutrition policy. While the Ralston et al study is focussed explicitly on food and nutrition, the issues and concepts addressed are relevant also to alcohol policy debates and present an important opportunity for shared learning across unhealthy commodity industries in order to protect and improve population health. This commentary addresses the importance of understanding how alcohol policy actors – especially decision-makers – perceive COI in relation to alcohol industry engagement in policy. A better understanding of such perceptions may help to inform the development of guidelines to identify, manage and protect against risks associated with COI in alcohol policy.

## Background

Ralston et al present an analysis of policy actor responses to a consultation on a draft World Health Organization (WHO) tool to prevent and manage conflicts of interest (COI) in nutrition policy.^1^ This proposed tool was designed to support Member States by offering a six-step risk assessment guide for use when considering engagement with non-state actors in developing and implementing nutrition policy.^1^ Ralston et al rightly highlight the importance of the issue of COI in global health and how involvement of commercial actors has “emerged as a central fault line in contemporary health governance” debates. This is especially true in efforts to tackle the global burden of non-communicable diseases given the role that consumption of health harming commodities such as tobacco, alcohol, processed foods and sugar-sweetened beverages – and the trans-national corporations which produce and market these – play in the aetiology of such conditions.^2^ The aim of the analysis conducted by Ralston et al was to explore how policy actors across sectors understand COI and the way they use this concept to frame the terms of engagement with the commercial sector. While Ralston et al focus on perceptions of COI in a food and nutrition policy context, the issues and concepts addressed are also relevant to alcohol policy debates where industry involvement is highly contested and has led to a number of policy failures. This commentary suggests that gaining a better understanding of how alcohol policy actors perceive industry involvement in policy could inform the development of guidelines to be used by decision-makers for identifying, managing and protecting against risks associated with COI.

###  Alcohol industry involvement in public policy is contested, but little guidance exists on managing COI in alcohol policy settings

The alcohol industry’s involvement and influence in health policy processes has been well-documented by researchers and has been subject to fierce criticism from the global public health community.^3^ In 2013, more than 500 researchers, practitioners and non-governmental organisation (NGO) representatives signed a joint statement of concern, calling on WHO to develop principles for managing COI related to alcohol industry involvement in the delivery of the WHO Global Strategy to reduce harmful use of alcohol.^4^ The involvement of alcohol industry actors in health policy initiatives has resulted in a number of prominent policy failures and stalemates. For example, the European Union’s (EU’s) Alcohol and Health Forum collapsed following the resignation of public health NGO members, who attributed a lack of progress in developing an EU Alcohol Strategy to industry interference.^5^ In the United Kingdom, the Public Health Responsibility Deal Alcohol Network was abandoned after public health representatives resigned, citing industry obstruction to the introduction of evidence-based policies such as minimum unit pricing.^6^ Alcohol industry influence in low-income country settings has also been highlighted as a cause for concern by researchers and advocates: evidence exists to demonstrate alcohol industry bodies drafted national alcohol strategies in a number of sub-Saharan African countries between 2006-2008. Analysis of these strategies found “the proposed policies serve the interests of industry at the expense of public health by attempting to enshrine ‘active participation of all levels of the beverage alcohol industry as a key partner in the policy formulation and implementation process.’”^7^ A 2018 systematic review by McCambridge and colleagues summarized the available evidence on alcohol industry involvement in policy-making and concluded that ‘alcohol industry actors are highly strategic, rhetorically sophisticated and well-organised in influencing national policy-making.’^3^

Despite the obvious consequences of industry engagement for the development of effective policy regimes, and calls from public health actors to protect alcohol policy from industry interference, to date no international guidelines have been developed on how to prevent and manage COI in relation to commercial sector engagement in alcohol policy. Guidelines exist in relation to accepting alcohol industry funding for research,^8,9^ and the WHO Framework of Engaging with Non-State Actors offers guidance for WHO officials engaging with private sector actors, however no criteria or principles for WHO relations with alcohol industry actors are specified.^10^ Furthermore, little published research has documented how policy actors – and in particular decision-makers – perceive COI and alcohol industry engagement. An in-depth understanding of how alcohol policy actors perceive COI in relation to alcohol industry engagement in health policy, drawing on insights derived from the investigation conducted by Ralston et al, could inform the development of guidelines for decision-makers to identify, manage and prevent COI in alcohol policy settings.

###  Commercial sector actors oppose restrictions on their engagement in policy processes and criticise public health actors for apparent vested interests

The analysis by Ralston et al indicates that commercial sector actors are highly engaged in nutrition policy debates and oppose restrictions to their activities. This high level of interest is illustrated by the comparable volume of responses from food industry bodies to the WHO consultation (n = 14) with NGO responses (n = 12). Given this high level of engagement, it is perhaps unsurprising that Ralston et al report that food industry actors strongly reject comparisons with the tobacco industry and to being excluded from policy processes. Alcohol industry bodies have similarly objected to parallels with ‘big tobacco’ in representations to governments,^11^ and have criticised calls to restrict alcohol industry engagement in ways to those set out in Article 5.3 of the WHO’s Framework Convention on Tobacco Control.^12^ Indeed, alcohol industry bodies have expressed criticism of the involvement of public health actors, in particular scholars and medical professionals, in policy processes, who they report hold vested interests in health policy outcomes which further their professional profiles, research funding opportunities and/or philosophical beliefs.^13-15^

In addressing COI in public policy settings, it is of course important for decision-makers to recognise the existence of a range of competing interests among and between different actors. However, a crucial distinction must be drawn between having an *interest* in a policy outcome and having a* conflict of interest* that has the potential to risk public health.^16^ The WHO nutrition tool, with its risk assessment approach, is an innovative policy instrument which allows for consideration of all interests presented by non-state actors in relation to their convergence with public health goals. The pragmatic approach adopted by the WHO nutrition tool represents an opportunity for decision-makers to distinguish between and help to manage competing *interests* and identify where *COI *emerge, that warrant further action due to their propensity to threaten or undermine public health goals. Ralston et al identified support for this pragmatic approach amongst public health actors, but opposition from food industry actors, who reported the tool unduly restricted private sector engagement in nutrition policy.^1^ Whilst it is likely that alcohol industry bodies would oppose restrictions on their engagement, little is known about how public health actors would view an approach that in principle accepted some forms of engagement with alcohol industry in policy settings was possible. In order to better manage COI in alcohol policy settings, further research is needed to explore public health actors’ perceptions of COI in relation to alcohol industry involvement in policy.

###  A more detailed typology of COI in alcohol policy settings is needed to move beyond a binary choice of engagement versus exclusion

 Within nutrition policy, Ralston et al identify mixed beliefs amongst public health actors relating to COI, with some perceiving the presence of COI to preclude any engagement in policy, whereas others purported that COI as a concept could be managed in order to mitigate risks to health policy outcomes. The authors call for a more detailed typology of COI that can be operationalised and applied in diverse policy contexts, which would also represent a departure from crude binary positions of engagement versus exclusion of commercial actors.

 It can be argued that alcohol policy decision-makers are currently presented with the challenge of navigating policy choices in the face of polarised arguments both for commercial sector partnership and commercial sector exclusion. The absence of guidelines to manage COI in alcohol policy settings may be hindering policy progress, both by enabling industry actors to obstruct evidence based public health measures and also because public health bodies refuse to engage with or support partnership initiatives involving industry.

As Ralston et al identify, an important aspect of the WHO nutrition tool is that it helps decision-makers to move beyond a binary approach to industry engagement, by acknowledging the nuances which exist in such policy settings and the opportunities for “restricted engagement” with non-state actors.^1^ Emulating the analysis of how nutrition policy decision-makers view the spectrum of risk associated with engaging industry in an alcohol policy context would provide valuable insights into the perceived benefits and/or disadvantages to involving alcohol industry actors according to decision-makers. Specifically, exploring how decision-makers’ views on alcohol industry involvement vary according to different policy scenarios, may inform the development of a risk assessment tool, similar to the WHO nutrition tool. Taking a systematic approach to assessing perceived risks linked to hypothetical situations linked to different types of industry actor, different policy topics, different engagement activities at various stages of the policy process would facilitate a greater understanding of how decision-makers view alcohol industry involvement in practice, as opposed to in the abstract.

In developing a more detailed typology of COI, Ralston et al call for a better definition of what constitutes the food industry, which, like the alcohol industry, is not a monolith.^17,18^ The authors highlight the food industry incorporates a diverse range of actors from community-based farming cooperatives to transnational corporations. Similarly, alcohol industry stakeholders range from pub landlords to multinational producers. These different corporate actors will have varying levels of interest in policy interventions and will be able to provide technical expertise of varying levels of relevance and utility to health policy. In assessing how the products, policies and practices of the actor seeking to engage in health policy aligns with the policy goals, and balancing this against what technical expertise the actor can contribute to the policy process, the WHO nutrition tool provides an important guide for decision-makers to navigate the diverse and multisectoral food industry.^19^ A more refined definition of the alcohol industry, including sub-categories of different actors, could assist decision-makers in assessing potential engagement with alcohol industry actors.

When assessing the utility of industry involvement in health policy processes it is essential that robust evaluation data is considered. Limited published research is available relating to evaluation of corporate social responsibility initiatives, which are often not subject to independent assessment.^3^ An analysis of more than 3500 alcohol industry corporate social responsibility actions across six global geographic regions found that the overwhelming majority (96.8%) initiatives lacked scientific support and 11% had the potential for doing harm.^20^ Lessons from the WHO nutrition tool, that includes consideration of the ‘technical impacts’ of an initiative within the risk assessment for engaging with commercial actors,^19^ could be applied to inform the development of a similar risk assessment for alcohol industry actors.

## Concluding Thoughts

Ralston et al conclude that effective health governance requires greater understanding of how COI can be conceptualised and managed amid high levels of contestation on policy engagement with commercial sector actors.^1^ Findings from this study indicate that investigating alcohol policy actor perceptions of COI would be an important exercise in progressing alcohol policy debates and seeking to rectify the policy failures which have characterised this field to date. Alcohol policy decision-makers are faced with calls to exclude industry from policy processes, yet alcohol companies continue to occupy prominent roles in health governance. Improving our understanding of how policy actors – especially decision-makers – perceive COI in relation to different industry stakeholders, and the potential contribution commercial organisations can make to advancing health goals, could help to inform the development of guidance for engaging with alcohol industry that meets the needs of decision-makers and aspires towards optimal public health outcomes.

## Ethical issues

 Not applicable.

## Competing interests

 KS reports the Institute of Alcohol Studies receives funding from the Alliance House Foundation.

## Author’s contribution

 KS is the single author of the paper.
